# The Transformer Bridge Principle Circuit Using RF Admittance Technology

**DOI:** 10.3390/s23125434

**Published:** 2023-06-08

**Authors:** Fanfan Liu, Chaojie Zhang, Wenyong Guo, Xinglong Pan

**Affiliations:** College of Power Engineering, Naval University of Engineering, Wuhan 430030, China

**Keywords:** radio frequency admittance, liquid level measurement, dirty, liquid level sensor

## Abstract

To investigate the problem of the lag stability of the capacitance value during the level drop of the dirty U-shaped liquid level sensor, the equivalent circuit of the dirty U-shaped liquid level sensor was analyzed, and the transformer bridge’s principle circuit that uses RF admittance technology was designed accordingly. Using the method of controlling a single variable, the measurement accuracy of the circuit was simulated when the dividing capacitance and the regulating capacitance had different values. Then, the right parameter values for the dividing capacitance and the regulating capacitance were found. On this basis, the change of the sensor output capacitance and the change of the length of the attached seawater mixture were controlled separately under the condition of removing the seawater mixture. The simulation outcomes showed that the measurement accuracy was excellent under various situations, validating the transformer principle bridge circuit’s efficacy in minimizing the influence of the output capacitance value’s lag stability.

## 1. Introduction

The prosperity and development of the marine industry put forward increasing requirements for the ship industry [1]. Additionally, the technical performance of ships is also steadily developing in the direction of automation [2]. As an important system to realize ship automation, the ship liquid level monitoring system is mainly used to monitor the liquid level in the ship’s seawater tank [3,4,5]. Seawater tank in ships is an important cabin room that adjusts the balance of the boat and improves manipulation, it plays a pivotal role in maintaining the stability of the ship and the draft of the ship, and so, realizing the accuracy and reliable measurement of the internal liquid level in the seawater tank is particularly important for ship’s sailing safety [6,7].

**Figure 1:**
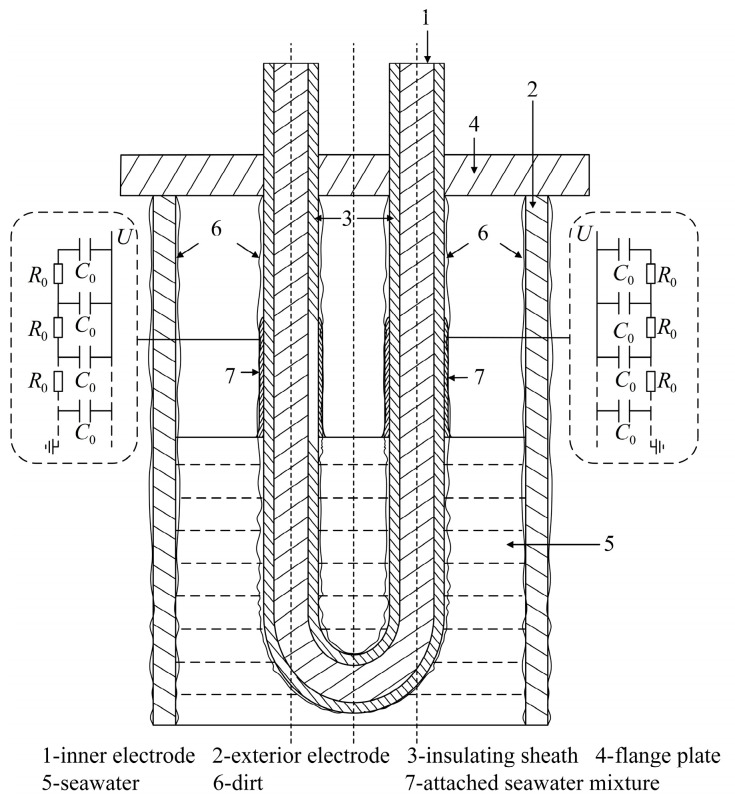
Schematic diagram of the U-shaped liquid level sensor with dirt.

The liquid level sensor is a piece of critical equipment for measuring the internal liquid level of seawater tanks [8]. Taking a certain type of ship as an example, the U-shaped liquid level sensor is a sort of capacitive liquid level sensor with a “U-shaped” body structure that is used to monitor the seawater level in the seawater tank. According to Figure 1, it consists primarily of inner electrode 1, exterior electrode 2, insulating sheath 3, and flange plate 4. Its fundamental principle is to measure the liquid level utilizing the mathematical relationship between electrical capacity and liquid level height.

In theory, the U-shaped liquid level sensors can measure the liquid level of seawater tanks accurately and reliably. However, the seawater tank’s environment is distinguished by excessive salinity and high humidity. The equipment parts and components that were exposed to this environment for an extended period of time will corrode, and the rust and sediment produced, the paint falling from the equipment surface, and small marine organisms will attach to the surface of the equipment in the tank as the seawater flows through the seawater tank. Solid sodium chloride precipitated from seawater will also collect on the surface of the apparatus when the liquid level is low. As the service life of the level sensor increases, the sensor cannot avoid this problem of dirt accumulation. This dirty mixture comprises primarily of sediment, rust, sodium chloride solid precipitated from seawater, microscopic marine creatures [6], and falling paint, which is apparent in dirt 6 in Figure 1. In the actual use process, it was determined that after the U-shaped liquid level sensor was installed for an extended period of time, the output capacitance value of the sensor will lag stabilize during the process of liquid level drop, i.e., the output capacitance value will require a period of time to gradually stabilize after the falling liquid level stabilized. It directly leads to the inability to measure the internal liquid level of seawater storage tanks quickly and accurately, and posing a certain safety hazard to the navigation of ships.

A thin layer of dirt will adhere to the insulating sheath’s surface. The surface of the dirt is uneven and porous, and the sea water itself is adhesive. During the decline of the liquid level, some sea water will penetrate the dirt or attach to its surface, resulting in the dirt’s continued conductivity up to a certain height above the liquid level. This portion of the seawater mixture has a high water content and sufficient ion mobility. The seawater mixture and external electrode are electrically conductive, resulting in a false liquid level and extra capacitance, resulting in a greater output capacitance of the sensor at this liquid level. However, as time passes, the seawater immersed in the dirty interior and adhered to the dirty surface will slowly slide down, the false liquid level will decrease, and the capacitance value will continue to decrease, leading to a lag stability of the capacitance value of the liquid level sensor during the liquid level drop. For ease of explanation, the combination of dirt, seawater, and seawater adhering to the outer surface within a specified height above the liquid level and generating a false liquid level is referred to as “attached seawater mixture,” represented in Figure 1 as an attached seawater mixture 7.

To reduce the impact of dirt on the lag stability of the U-shaped liquid level sensor’s capacitance value, radio frequency admittance capacitive liquid level measuring technology will be used. On the basis of the radio frequency admittance theorem, the effect of the attached seawater mixture will be investigated.

The radio frequency admittance technique is presented in order to address the problem of latency and stability of capacitance value in a U-shaped liquid level sensor. Nowadays, the capacitive radio frequency admittance liquid level sensor based on this technology covers almost all measurements of medium liquid level [9,10,11,12,13,14]. Heywood N. et al. investigated and concluded that the capacitive radio frequency admittance liquid level sensor has the benefits of a broad measurement range, high measurement accuracy, a big limit pressure, a large working temperature range, and no effect from hanging materials [15]. Chen Wei of Nanjing Forestry University and collaborators created a radio frequency admittance capacitance level meter that enables remote liquid level real-time monitoring through the combination of virtual instrument software LabVIEW and network technologies [16]. The digital radio frequency admittance capacitive liquid level sensor created by Yu Nan and colleagues at Huaiyin Institute of Technology is network-enabled thanks to their implementation of TCP/IP communication protocol and TCP socket communication [17]. In terms of sensor application, Zhou Liang and others developed the KRF series radio frequency admittance liquid level meter in response to the need for accurate measurement of liquid level in the petrochemical industry. This instrument effectively resolved the issue of false liquid level in the measurement process of capacitive liquid level sensors, and the measurement accuracy reached 0.5% [18]. Fan Xue and coworkers devised a radio frequency admittance capacitive liquid level monitoring system for the fermentation process of large yellow rice wine that showed excellent measurement accuracy and was unaffected by hanging objects. Using RFID technology, the system enabled wireless communication between the fermentation tank and the fermentation tank, as well as between the fermentation tank and the monitoring system [19].

The signal acquisition and processing procedure of radio frequency admittance liquid level measuring technology is predicated primarily on the fact that the phase of the phase current flowing through the measured liquid capacitance precedes the phase of the phase current flowing through the hanging layer. Zhang Chao of Shanghai Jiaotong University and coworkers used software to delay the interrupt service program of the PIC18F452 single-chip microcontroller, and then delayed the phase difference before initiating the A/D collector to sample [20]. Chen Xiaozhu of the Chinese Institute of Metrology and coworkers filtered the amplitude signal of phase time using the MAX903 comparator, and then performed A/D conversion for the square wave after phase shift [21]. There were flaws in this strategy as well. Due to the high frequency of the RF signal, the phase time of the signal was very short. The signal-to-noise ratio of the signal generated by this approach was low, and the anti-interference capacity was weak, but this was necessary if you want to sample during the time of falling behind the phase.

In order to enhance the signal-to-noise ratio of the output signal, consecutively, the fixed-point sampling integration technique and the chopping time-domain integration method were presented. The technique of sampling at the phase point at intervals of one or more cycles and conducting cumulative averaging is referred to as the fixed-point sampling integration method. This strategy lowers the influence of noise to a certain degree and reduces the conversion speed requirements [22,23,24]. Chopping time-domain integration technique refers to the method of chopping the signal and then integrating the signal in time domain within the phase range, which can effectively minimize the effects of the hanging layer interference signal, and may more effectively increase the signal-to-noise ratio of the output signal, therefore facilitating the future signal processing [24].

To investigate the problem of the lag stability of the capacitance value during the level drop of the dirty U-shaped liquid level sensor, and reducing the impact of the attached seawater mixture, the rest of this paper is organized as follows. Section 2 analyzes the equivalent circuit of the dirty U-shaped liquid level sensor. The transformer bridge’s principle circuit that uses RF admittance technology is designed in Section 3. Additionally, the establishment of simulation circuit model of transformer bridge principle circuit is given in Section 4. In Section 5, using the method of controlling a single variable, the measurement accuracy of the circuit is simulated when the dividing capacitance and the regulating capacitance have different values. Additionally, Section 6 mainly conducted simulation on the transformer bridge principle circuit, the effectiveness of the signal processing circuit in reducing the influence of output capacitance hysteresis stability was verified. Section 7 concludes the paper.

## 2. Equivalent Circuit Analysis of U-Shaped Liquid Level Sensor with Dirt

The size of the circuit’s electrical components and the wavelength of the working signal determine whether the circuit is a lumped parameter circuit or a distributed parameter circuit. The ratio of the speed of light in a vacuum to the frequency of the working signal is equal to the wavelength of the signal being produced by the working device. A lumped parameter circuit is one in which the electrical device size is much less than the wavelength. It is characterized by the fact that the size and location of a device have no effect on the voltage between any two terminals in the circuit or the current flowing into the device. If the size of the circuit’s electrical components does not match the criterion of being much smaller than the wavelength, the circuit is known as a dispersed parameter circuit. It is characterized by the fact that the voltage and current in the circuit are proportional to time, geometric size, and spatial location.

The term “radio frequency” (RF) is used to denote the frequency range of very high frequency radio waves, and “radio frequency admittance” (RF admittance) describes the method of measuring admittance using such a radio frequency range. An impedance *Z* is a complex quantity whose reciprocal is the admittance *Y*. Resistance *R* is the real quantity, whereas reactance *X* is the imaginary quantity. Each of the three components of an impedance circuit—resistance (*R*), capacitance (*C*), and inductance (*L*)—contributes to the total impedance (*Z*). The equivalent impedance of the circuit when the three are connected in series is [25]:

(1) $$Z=R+jX=R+j\left(\omega L-\frac{1}{\omega C}\right)$$

Admittance is [25]

(2) $$Y=\frac{1}{Z}=\frac{1}{R+jX}=\frac{R}{R^{2}+X^{2}}+j\left(\frac{-X}{R^{2}+X^{2}}\right)=G+jB$$

Equation (2) shows that the admittance formed by the parallel connection of conductance *G* and admittance *B* is equivalent to the impedance formed by the series connection of resistance *R* and reactance *X*.

A schematic of a dirty U-shaped liquid level sensor is shown in Figure 1. A sinusoidal RF excitation voltage $\overset{\cdot }{U}$ with angular frequency ω is applied between the inner and outer electrodes of the U-shaped liquid level sensor. The inner side of the insulating sheath is directly linked to the inner electrode, while the outside side is connected to the outer electrode through the seawater being measured. From an electrical point of view, since the liquid level sensor works with radio frequency, the size of the attached seawater mixture does not reach the condition of being much smaller than the wavelength of the radio frequency signal. This means that the attached seawater mixture should be processed using the distributed parameter circuit.

The dry dirt may be considered uniform, and the attached seawater mixture with a false liquid level contains a substantial amount of water. Owing to the huge cross-sectional area of the sea water measured below the liquid level in the seawater tank, the resistance of this part of sea water may be regarded to be around 0. In contrast, the attached seawater mixture is merely a thin layer adhered to the surface of the insulating sheath, with a very small cross-sectional area, a very high equivalent resistance, and a negligible inductance. Furthermore, the insulation of the insulating sheath is excellent; thus, the leakage conductance between the inner electrode and the associated seawater mixture may be disregarded. The sensor uses an RF frequency to function. The attached seawater mixture on the left and right cables may be approximated as a transmission line made of infinite resistance micro-elements $R_{0}$ and capacitance micro-elements $C_{0}$, where the resistance micro-elements $R_{0}$ represent the unit length resistance of the seawater mixture attached to the left and right cables along the direction of the insulating sheath, and the capacitance micro-elements $C_{0}$ represent the unit length capacitance of the insulating sheath as dielectric.

Take the single-sided cable as the topic of study and record the unit length series impedance of the equivalent transmission line as $Z_{0}$, ignoring the inductance, $Z_{0}$ can be expressed as [26]

(3) $$Z_{0}=R_{0}+j\omega L=\frac{\rho }{S}\;$$

where ω is the angular frequency, L is the inductance per unit length of the equivalent transmission line and L=0, ρ is the resistivity of the attached mixture of seawater, and S is the cross-sectional area of the attached seawater mixture.

Note that the parallel admittance per unit length of the equivalent transmission line is $Y_{0}$, since the leakage conductance is ignored, $Y_{0}$ can be expressed as [26]

(4) $$Y_{0}=G+j\omega C_{0}=j2\pi fC_{0}$$

where G is the corresponding transmission line’s leakage current per unit length G=0 and f is its frequency.

The impedance of the equivalent transmission line can be expressed as [26]

(5) $$Z_{eql}=\sqrt{\frac{Z_{0}}{Y_{0}}}$$

Substitute Formulas (3) and (4) into Formula (5) and simplify it to

(6) $$Z_{eql}=\frac{1-j}{2}\sqrt{\frac{\rho }{S\pi fC_{0}}\;}=\sqrt{\frac{\rho }{2S\pi fC_{0}}\;}∠\left(-\frac{\pi }{4}\right)$$

when the length of the attached seawater mixture is enough, the seawater mixture that is attached to one side of the cable may approximately be considered as a transmission line. This occurs when the length of the attached seawater mixture is sufficient. According to Formula (6), the absolute values of the real and imaginary parts of the equivalent impedance of the attached seawater mixture on one side of the cable are equal. This indicates that the modulus of the equivalent resistance and the equivalent capacitance are also equal. The equivalent resistance can be understood as the pure resistance of the attached seawater mixture. The equivalent capacitance can be understood as the pure capacitance of the equivalent resistance. The capacitance that may be regarded as the equivalent capacitance is the capacitance that exists between the attached seawater mixture and the inner electrode, with the insulating sheath serving as the dielectric.

**Figure 2:**
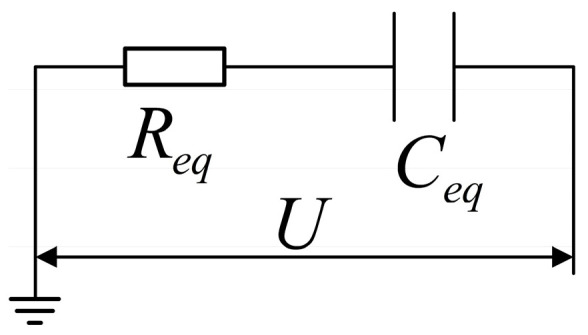
Equivalent circuit of attached seawater mixture to single-side cable.

Due to the presence of an insulating sheath between the inner electrode and the seawater used in the measurement, no direct current channel exists between the inner electrode and the earth. As can be seen in Figure 2, from an electrical perspective the impedance of the seawater mixture attached to the single-side cable may be comparable to a resistance $R_{eq}$ and a capacitance $C_{eq}$ in series, and the impedance of the capacitance $C_{eq}$ is recorded as $Z_{C}$.

(7) $$R_{eq}=\sqrt{\frac{\rho }{2S\pi fC_{0}}\;}\cos\left(-\frac{\pi }{4}\right)=\frac{1}{2}\sqrt{\frac{\rho }{2S\pi fC_{0}}\;}$$

(8) $$Z_{C}=-\frac{j}{\omega C_{eq}}=\sqrt{\frac{\rho }{2S\pi fC_{0}}\;}j\sin\left(-\frac{\pi }{4}\right)=-\frac{j}{2}\sqrt{\frac{\rho }{2S\pi fC_{0}}\;}$$

(9) $$Z_{eq}=R_{eq}+Z_{C}=R_{eq}-\frac{j}{\omega C_{eq}}$$

**Figure 3:**
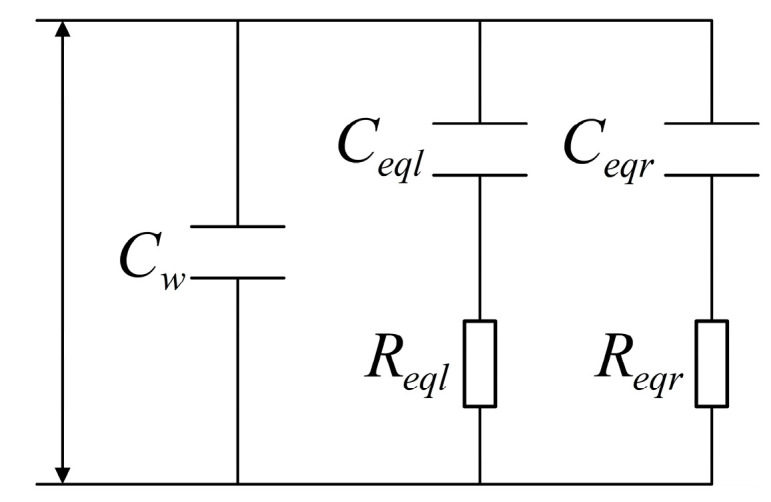
Circuit model of the U-shaped structure liquid level sensor with dirt.

It is important to note that $R_{eql}$ and $C_{eql}$ represent the equivalent resistance and capacitance of the seawater mixture attached to the left cable, whereas $R_{eqr}$ and $C_{eqr}$ indicate the equivalent resistance and capacitance of the seawater mixture tied to the right cable. From an electrical perspective, the attached seawater mixture on both sides of the cables, which influences the lag stability of the sensor capacitance value, may be compared to a circuit made up of capacitance $C_{eql}$ and $R_{eql}$ resistance, capacitance $C_{eqr}$ and resistance $R_{eqr}$. Figure 3 shows the circuit model of the dirty U-shaped liquid level sensor. The inner and outer electrodes of the U-shaped liquid level sensor are the equivalent circuit poles of the seawater mixture linked to both sides of the wires.

## 3. Principle Circuit Design of Transformer Bridge

**Figure 4:**
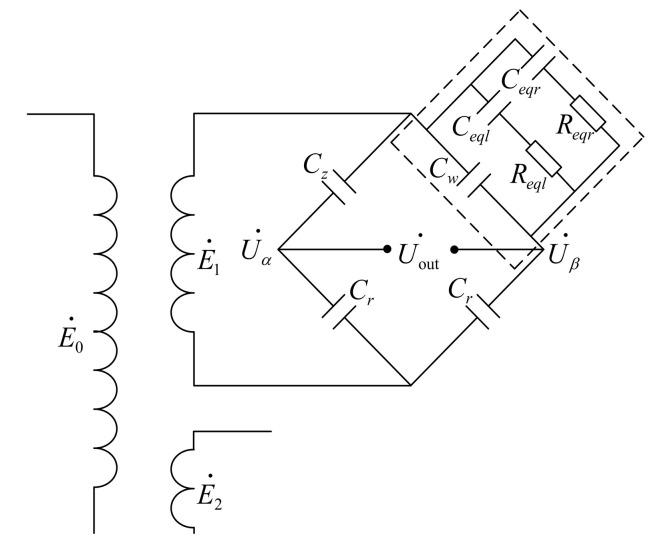
Transformer bridge principle circuit.

Figure 4 depicts the transformer bridge circuit used to reduce the effect of capacitance value’s lag stability during the level drop of the dirty U-shaped liquid level sensor. This circuit transmits the sinusoidal AC signal to the bridge circuit via the transformer, detects the sensor capacitance under the condition of removing the attached seawater mixture via the bridge circuit, collects the output voltage signal from the bridge circuit, performs signal fitting processing, and inputs the fitted signal function into the corresponding program.

The architecture that involves sending sinusoidal alternating current signals to the bridge circuit through transformer has two benefits. The design has the potential to increase the measurement range of capacitance on the one hand, while on the other, the transformer has the ability to give synchronous sampling trigger signal. The transformation ratio of the transformer is n, and the impedance of the secondary equivalent load may be altered to $1/n^{2}$ that of the primary impedance by utilizing the transformer as opposed to adding the input signal directly to the bridge circuit while maintaining the same load at the input end.

Using a transformer with a transformation ratio greater than one may effectively lower the impedance of the secondary equivalent load, while maintaining the input power and rated power of the transformer, so as to increase the measuring range of the liquid level capacitance. Nevertheless, a greater input voltage may be produced through the transformer when the capacitance detection range of the liquid level sensor is fixed. In addition, synchronous sampling signals $\overset{\cdot }{E_{2}}$ may be generated with the use of a transformer. Add a sinusoidal AC voltage with an angular frequency of ω to the primary side of the transformer, record $\overset{\cdot }{E_{0}}$ as the phase voltage of the primary side of the transformer, record $\overset{\cdot }{E_{1}}$ and $\overset{\cdot }{E_{2}}$ as the phase voltage of the two secondary sides of the transformer, where $\overset{\cdot }{E_{2}}$ is the synchronous phase voltage with the same frequency as the phase voltage $\overset{\cdot }{E_{1}}$, whose function is to provide the required sampling trigger signal for a particular time after a series of processing.

The adjusting capacitance $C_{r}$, as illustrated in Figure 4, is the capacitance connected to the range, and its value is controlled by the sensor range. $C_{z}$ is the dividing capacitance, $C_{r}$ and $C_{z}$ shared it with the phase voltage $\overset{\cdot }{E_{1}}$. $C_{w}$ is the sensor’s output capacitance after removing the associated seawater mixture. The circuit is built on the unbalanced bridge idea. When the output capacitance $C_{w}$ varies due to the selection of suitable $C_{r}$ and $C_{z}$ values, the bridge circuit is in an imbalanced condition. Hence, the signal $\overset{\cdot }{U_{\mathrm{out}}}$ comprising the relevant attributes of $C_{w}$ is output. The capacitance value’s lag stability of the U-shaped liquid level sensor may be reduced with specialized processing, and the value of the output capacitance $C_{w}$ can be achieved. The whole procedure is as follows.

Figure 4 shows the equivalent circuit of the U-shaped liquid level sensor’s output capacitance for dirt, represented by the dashed box; the total equivalent circuit impedance of the output capacitance is denoted by $Z_{\sigma }$.

(10) $$Z_{\sigma }=\frac{1}{j\omega C_{\omega }+\frac{1}{Z_{eql}}+\frac{1}{Z_{eqr}}}$$

The equivalent impedance of the seawater mixture in the left and right cables is $Z_{eql}$ and $Z_{eqr}$, respectively. Formula (1) may be used to determine the equivalent impedance of the seawater mixture when it is connected to both left and right wires.

(11) $$Z_{eql}=R_{eql}-\frac{j}{\omega C_{eql}}$$

(12) $$Z_{eqr}=R_{eqr}-\frac{j}{\omega C_{eqr}}$$

According to the radio frequency admittance theorem, the real part and the imaginary part of the equivalent impedance of the seawater mixture attached to the cable on one side have the same absolute value. These absolute values are equal.

(13) $$R_{eql}=\frac{1}{\omega C_{eql}}$$

(14) $$R_{eqr}=\frac{1}{\omega C_{eqr}}$$

Substitute Formula (13) into Formula (11)

(15) $$Z_{eql}=R_{eql}-\frac{j}{\omega C_{eql}}=\frac{1-j}{\omega C_{eql}}$$

Similarly, substituting Formula (14) into Formula (11)

(16) $$Z_{eqr}=R_{eqr}-\frac{j}{\omega C_{eqr}}=\frac{1-j}{\omega C_{eqr}}$$

Substitute Formulas (15) and (16) into Formula (10)

(17) $$Z_{\sigma }=\frac{1-j}{\left(1+j\right)\omega C_{w}+\omega \left(C_{eql}+C_{eqr}\right)}$$

In Figure 4, $\overset{\cdot }{U_{\alpha }}$ and $\overset{\cdot }{U_{\beta }}$ respectively represent the phase voltage on both sides of the sensor output capacitance when the dividing capacitance $C_{z}$ and the attached seawater mixture are removed, and $\overset{\cdot }{U_{\mathrm{out}}}$ represents the output voltage. The relationship between the three is $\overset{\cdot }{U_{\mathrm{out}}}=\overset{\cdot }{U_{\alpha }}-\overset{\cdot }{U_{\beta }}$.$\overset{\cdot }{U_{\alpha }}$ and $\overset{\cdot }{U_{\beta }}$ can be expressed as

(18) $$\overset{\cdot }{U_{\alpha }}=\frac{1/j\omega C_{z}}{1/j\omega C_{z}+1/j\omega C_{r}}\overset{\cdot }{E_{1}}=\frac{C_{r}}{C_{z}+C_{r}}\overset{\cdot }{E_{1}}$$

(19) $$\overset{\cdot }{U_{\beta }}=\frac{Z_{\sigma }}{Z_{\sigma }+1/j\omega C_{r}}\overset{\cdot }{E_{1}}$$

Substitute Formula (17) into Formula (19)

(20) $$\overset{\cdot }{U_{\beta }}=\frac{\left(1+j\right)C_{r}}{\left(1+j\right)\left(C_{w}+C_{r}\right)+C_{eql}+C_{eqr}}\overset{\cdot }{E_{1}}$$

Substitute Formulas (18) and (20) into $\overset{\cdot }{U_{\mathrm{out}}}=\overset{\cdot }{U_{\alpha }}-\overset{\cdot }{U_{\beta }}$

(21) $$\begin{array}{l} \overset{\cdot }{U_{\mathrm{out}}}=\frac{C_{r}}{C_{z}+C_{r}}\overset{\cdot }{E_{1}}-\frac{\left(1+j\right)C_{r}}{\left(1+j\right)\left(C_{w}+C_{r}\right)+C_{eql}+C_{eqr}}\overset{\cdot }{E_{1}}\\ =\frac{\left(1-j\right)C_{r}\left(C_{eql}+C_{eqr}\right)+2\left(C_{w}-C_{z}\right)C_{r}}{\left(C_{z}+C_{r}\right)\left[2\left(C_{w}+C_{r}\right)+\left(1-j\right)\left(C_{eql}+C_{eqr}\right)\right]}\overset{\cdot }{E_{1}} \end{array}$$

The capacitors $C_{eql}$ and $C_{eqr}$ take the insulating sheath as the dielectric, select the appropriate adjusting capacitor $C_{r}$, so that the formula $C_{w}+C_{r}\gg C_{eql}+C_{eqr}$ is established, so the Formula (21) can be simplified as

(22) $$\begin{array}{l} \overset{\cdot }{U_{\mathrm{out}}}=\frac{\left(1-j\right)C_{r}\left(C_{eql}+C_{eqr}\right)+2\left(C_{w}-C_{z}\right)C_{r}}{2\left(C_{z}+C_{r}\right)\left(C_{w}+C_{r}\right)}\overset{\cdot }{E_{1}}\\ =\frac{\left(C_{w}-C_{z}\right)C_{r}}{\left(C_{z}+C_{r}\right)\left(C_{w}+C_{r}\right)}\overset{\cdot }{E_{1}}+\frac{\left(1-j\right)C_{r}\left(C_{eql}+C_{eqr}\right)}{2\left(C_{z}+C_{r}\right)\left(C_{w}+C_{r}\right)}\overset{\cdot }{E_{1}} \end{array}$$

If the secondary AC voltage of the transformer is $e_{1}\left(t\right)=E_{1}\cos\left(\omega t\right)$, then $\overset{\cdot }{E_{1}}=E_{1}e^{j0}$

(23) $$\overset{\cdot }{U_{\mathrm{out}}}=\frac{\left(C_{w}-C_{z}\right)C_{r}}{\left(C_{z}+C_{r}\right)\left(C_{w}+C_{r}\right)}E_{1}e^{j0}+\frac{C_{r}\left(C_{eql}+C_{eqr}\right)}{\sqrt{2}\left(C_{z}+C_{r}\right)\left(C_{w}+C_{r}\right)}E_{1}e^{j(-\frac{\pi }{4})}$$

In the formula, record

(24) $$\overset{\cdot }{U_{\sigma }}=\frac{\left(C_{w}-C_{z}\right)C_{r}}{\left(C_{z}+C_{r}\right)\left(C_{w}+C_{r}\right)}E_{1}e^{j0}$$

(25) $$\overset{\cdot }{U_{\lambda }}=\frac{C_{r}\left(C_{eql}+C_{eqr}\right)}{\sqrt{2}\left(C_{z}+C_{r}\right)\left(C_{w}+C_{r}\right)}E_{1}e^{j(-\frac{\pi }{4})}$$

Therefore, the corresponding sinusoidal quantities of phase voltages $\overset{\cdot }{U_{\sigma }}$,$\overset{\cdot }{U_{\lambda }}$ and $\overset{\cdot }{U_{\mathrm{out}}}$ can be expressed as

(26) $$u_{\sigma }\left(t\right)=\frac{\left(C_{w}-C_{z}\right)C_{r}}{\left(C_{z}+C_{r}\right)\left(C_{w}+C_{r}\right)}E_{1}\cos\left(\omega t\right)$$

(27) $$u_{\lambda }\left(t\right)=\frac{C_{r}\left(C_{eql}+C_{eqr}\right)}{\sqrt{2}\left(C_{z}+C_{r}\right)\left(C_{w}+C_{r}\right)}E_{1}\cos\left(\omega t-\frac{\pi }{4}\right)$$

(28) $$u_{\mathrm{out}}\left(t\right)=\frac{\left(C_{w}-C_{z}\right)C_{r}}{\left(C_{z}+C_{r}\right)\left(C_{w}+C_{r}\right)}E_{1}\cos\left(\omega t\right)+\frac{C_{r}\left(C_{eql}+C_{eqr}\right)}{\sqrt{2}\left(C_{z}+C_{r}\right)\left(C_{w}+C_{r}\right)}E_{1}\cos\left(\omega t-\frac{\pi }{4}\right)$$

**Figure 5:**
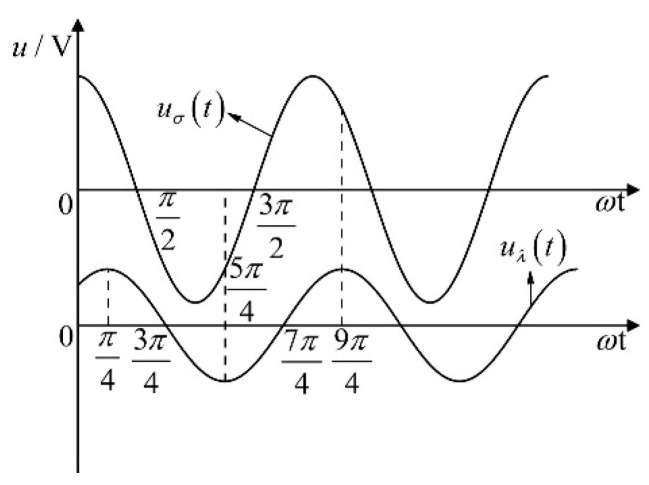
Phase diagram of the $u_{\sigma }\left(t\right)$ and $u_{\lambda }\left(t\right)$.

Figure 5 demonstrates that the phase of $u_{\lambda }\left(t\right)$ lags behind the phase of $u_{\sigma }\left(t\right)$ by π/4, where the voltage $u_{\sigma }\left(t\right)$ is only related to the output capacitance $C_{w}$ of the sensor when the attached seawater mixture is removed and it has nothing to do with the equivalent capacitance $C_{eql}$ of the seawater mixture attached to the left cable or the equivalent capacitance $C_{eqr}$ of the seawater mixture attached to the right cable. The voltage $u_{\lambda }\left(t\right)$ is related to the capacitances $C_{eql}$ and $C_{eqr}$. The output voltage $u_{\mathrm{out}}\left(t\right)$ results from the interaction between $u_{\sigma }\left(t\right)$ and $u_{\lambda }\left(t\right)$. To remove the impact of the equivalent capacitance $C_{eql}$ and $C_{eqr}$ on the output voltage $u_{\mathrm{out}}\left(t\right)$, just set the output voltage $u_{\lambda }\left(t\right)$ to zero. In Figure 5, the change curve of voltage $u_{\lambda }\left(t\right)$ with phase ωt shows that voltage $u_{\lambda }\left(t\right)$ is a periodic function with horizontal axis symmetry. Choose a suitable phase interval for voltage $u_{\lambda }\left(t\right)$ integration such that the integral value of voltage $u_{\lambda }\left(t\right)$ in this phase interval is zero.

Take phase interval π/4−5π/4 as an example, as seen in Figure 5, when ωt is in phase interval π/4−5π/4, the integral results of voltage $u_{\sigma }\left(t\right)$ and voltage $u_{\lambda }\left(t\right)$ are as follows.

(29) $$U_{\sigma }=\frac{\left(C_{w}-C_{z}\right)C_{r}E_{1}}{\left(C_{z}+C_{r}\right)\left(C_{w}+C_{r}\right)}\int_{\pi /4}^{5\pi /4}\cos\left(\omega t\right)\cdot d\omega t=-\frac{\sqrt{2}\left(C_{w}-C_{z}\right)C_{r}E_{1}}{\left(C_{z}+C_{r}\right)\left(C_{w}+C_{r}\right)}$$

(30) $$U_{\lambda }=\frac{C_{r}\left(C_{eql}+C_{eqr}\right)}{\sqrt{2}\left(C_{z}+C_{r}\right)\left(C_{w}+C_{r}\right)}\int_{\pi /4}^{5\pi /4}\cos\left(\omega t-\frac{\pi }{4}\right)\cdot d\omega t=0$$

The sampling interval signal is sampled and integrated during the first cycle π/4−5π/4. The definitive integral value of voltage $u_{\lambda }\left(t\right)$ is zero, removing the effect of equivalent capacitance $C_{eql}$ and $C_{eqr}$ on output voltage $u_{\mathrm{out}}\left(t\right)$, and recording the output voltage of transformer bridge circuit as $U_{\sigma \mathrm{out}}$.

(31) $$U_{\sigma \mathrm{out}}=-\frac{\sqrt{2}\left(C_{w}-C_{z}\right)C_{r}E_{1}}{\left(C_{z}+C_{r}\right)\left(C_{w}+C_{r}\right)}$$

Both $C_{z}$ and $C_{r}$ in Formula (31) are known, and the output capacitance $C_{w}$ of the U-shaped liquid level sensor when the attached seawater mixture is removed can be calculated.

(32) $$C_{w}=\frac{\left(\sqrt{2}E_{1}-U_{\sigma \mathrm{out}}\right)C_{r}C_{z}-U_{\sigma \mathrm{out}}C_{r}^{2}}{U_{\sigma \mathrm{out}}\left(C_{z}+C_{r}\right)+\sqrt{2}E_{1}C_{r}}$$

## 4. The Establishment of Simulation Circuit Model of Transformer Bridge Principle Circuit

**Figure 6:**
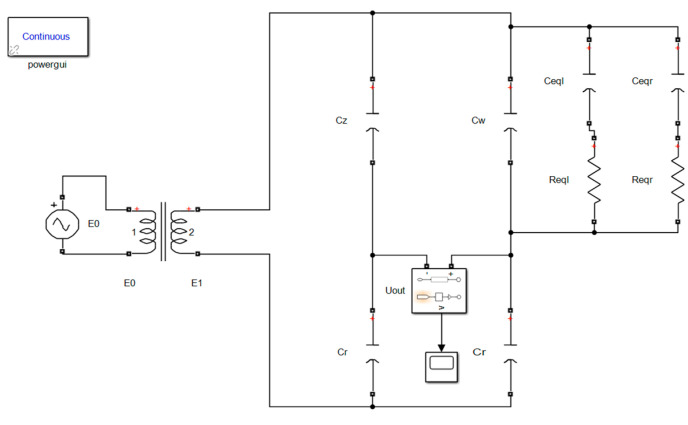
Simulation circuit model.

The simulation model of the transformer bridge principle circuit is built by using the MATLAB Simulink program, as shown in Figure 6. Then, the value of the regulating capacitance $C_{r}$ and the dividing capacitance capacitor $C_{z}$ is simulated, and finally, the transformer bridge principle circuit is tested. The primary concept behind this simulation is as follows: after the values of the parameters in the circuit have been determined, the output signal $u_{\mathrm{out}}\left(t\right)$ from the transformer bridge principle circuit will alter depending on the capacitance $C_{w}$ value. In accordance with Formulae (29)–(31), the voltage value $U_{\sigma \mathrm{out}}$ exclusively linked to capacitance $C_{w}$ may be derived by sampling and integrating the signal during π/4−5π/4. To acquire the voltage value $U_{\sigma \mathrm{out}}$ corresponding to distinct output signals $u_{\mathrm{out}}\left(t\right)$, first fit the different output signals $u_{\mathrm{out}}\left(t\right)$ to obtain the function expression of the output signal $u_{\mathrm{out}}\left(t\right)$, then complete the definite integral solution of the output signal function $u_{\mathrm{out}}\left(t\right)$ in the phase interval π/4−5π/4 via the program, and then calculate the capacitance $C_{w}$ according to Formula (32).

When an AC power source is used to input a sine AC signal $\overset{\cdot }{E_{0}}$, the signal frequency is f, and its relationship with angular frequency ω is ω=2πf. Both the main and secondary alternating current signals from the transformer may be represented as follows.

(33) $$e_{0}\left(t\right)=E_{0}\cos\left(\omega t\right)$$

(34) $$e_{1}\left(t\right)=E_{1}\cos\left(\omega t\right)$$

The input signal $\overset{\cdot }{E_{0}}$ provides a voltage signal to the bridge circuit through the transformer, and the selection of its frequency f is important. When the frequency of input signal $\overset{\cdot }{E_{0}}$ is too low, the wavelength of the signal is large, and the size of the attached seawater mixture is much smaller than the wavelength of the signal, then the attached seawater mixture will not be processed according to the distributed parameter circuit, and the circuit design of the transformer bridge principle will not be established. Conversely, when the frequency f of input signal is too high, it will also increase the difficulty of subsequent sampling. According to the frequency of the applicable RF admittance capacitance instrument, the input signal frequency chosen for this design is f=300 kHz and the angular frequency is $\omega =2\pi f=1.885\times 10^{6}\;\mathrm{rad}/s$.

As the AC voltage source, the common 24V AC voltage source is utilized. In accordance with the transformer’s impedance transformation principle, the secondary impedance is equal to $1/n^{2}$ of the primary impedance. Without modifying the input voltage or rated power, a transformer with a transformation ratio larger than 1 is used. The secondary impedance of the transformer is decreased, which can significantly expand the capacitance measurement range. In this design, the common transformer with a 2:1 transformation ratio is used; hence, the secondary voltage amplitude of the transformer is $E_{1}=12\;V$.

Equation (32) is obtained by neglecting tiny capacitance $C_{eql}$ and $C_{eqr}$, so when selecting capacitance $C_{r}$, equation $C_{w}+C_{r}$$\gg C_{eql}+C_{eqr}$ must be established. According to Formula (28), the output signal $u_{\mathrm{out}}\left(t\right)$ can be expressed as

(35) $$u_{\mathrm{out}}\left(t\right)=X\cos\left(\omega t\right)+Y\cos\left(\omega t-\frac{\pi }{4}\right)$$

(36) $$X=\frac{\left(C_{w}-C_{z}\right)C_{r}}{\left(C_{z}+C_{r}\right)\left(C_{w}+C_{r}\right)}E_{1}$$

(37) $$Y=\frac{C_{r}\left(C_{eql}+C_{eqr}\right)}{\sqrt{2}\left(C_{z}+C_{r}\right)\left(C_{w}+C_{r}\right)}E_{1}$$

Equation (35) can be simplified as

(38) $$u_{\mathrm{out}}\left(t\right)=\left(X+\frac{\sqrt{2}}{2}Y\right)\cos\left(\omega t\right)+\frac{\sqrt{2}}{2}Y\sin\left(\omega t\right)$$

Using MATLAB to analyze and fit the output signal $\overset{\cdot }{u_{\mathrm{out}}}\left(t\right)$ under various situations. According to Formula (38), function $u_{\mathrm{out}}\left(t\right)$ may be seen as the Fourier expansion with the first term equal to 0 and the series equal to 1, hence the Fourier model with the series equal to 1 is chosen as the fitting model, and the fitting equation is $y=a_{0}+a_{1}\cos(\omega t)+b_{1}\sin(\omega t)$.

The function expression of the output signal $u_{\mathrm{out}}\left(t\right)$ generated by fitting is swapped into the prepared MATLAB program, and the result $C_{w}$ is ultimately produced. The purpose of this program is to integrate the output signal $u_{\mathrm{out}}\left(t\right)$ in the phase interval π/4−5π/4 and determine the value of $C_{w}$ according to the Formula (32). Below is the calculating method for:

syms *t* % Define time *t* ω=2*pi*300,000;
% Angular frequency
*f*=a0+a1*cos(ω*t)+b1*sin(ω*t;
% Substitute the output signal function expression obtained by fitting
*a*=ω*int(f,t,pi/(4*ω),5*pi/(4*ω));
% integrate the output signal at π/4−5π/4
, and set the integral value as *a*
*b* = *Cr*;
% Enter the value of regulating capacitance
*c* = *Cz*;
% Enter the value of dividing capacitance *d* = ((12 * sqrt (2) − *a*) * *b* * *c* − *a* * *b* * *b*)/ (*a* * (*b* + *c*) + 12 * sqrt (2) * *b*);
$\%\;d\;\mathrm{represents}\;\mathrm{the}\;\mathrm{value}\;\mathrm{of}\;C_{w}$

## 5. Simulation Analysis of Transformer Bridge Principle Circuit Parameters $C_{z}$ and $C_{r}$

### 5.1. The Influence of the Value of Dividing Capacitance $C_{z}$ on the Measurement Accuracy of Capacitance $C_{w}$

The dividing capacitance $C_{z}$ and regulating capacitance $C_{r}$ values will be calculated using a single variable approach. Capacitance $C_{w}$ and the length of the attached seawater mixture will be kept constant, while only the dividing capacitance $C_{z}$ and the regulating capacitance $C_{r}$ will be regulated to vary at once. This will ensure that the capacitance $C_{eql}$, capacitance $C_{eqr}$, electrical resistance $R_{eql}$, and electrical resistance $R_{eqr}$ remain unchanged. Analyze the deviation from the theoretical value of $C_{w}$ in your simulations to settle on a final value for the transformer bridge principle circuit’s regulating capacitance $C_{r}$ and dividing capacitance $C_{z}$. The detection capacitance target range of the transformer bridge principle circuit is designed to be 0−2000.0 pF based on the change range of the output capacitance value of the U-shaped liquid level sensor without dirt. So the intermediate value of the target range, 1000 pF, is chosen as the theoretical value of $C_{w}$ during simulation.

The length of the seawater mixture attached to the left and right cables of the U-shaped liquid level sensor is $l_{l}$ and $l_{r}$ respectively. According to the capacitance formula of the coaxial cylindrical capacitor, the equivalent capacitance $C_{eql}$ or $C_{eqr}$ corresponding to the seawater mixture attached to the single side cable can be obtained as

(39) $$C_{eql}=\frac{2\pi \varepsilon_{0}\varepsilon_{F}l_{l}}{\ln\frac{R_{2}}{R_{1}}}$$

(40) $$C_{eqr}=\frac{2\pi \varepsilon_{0}\varepsilon_{F}l_{r}}{\ln\frac{R_{2}}{R_{1}}}$$

Substitute Formulas (39) and (40) into Formulas (13) and (14), respectively.

(41) $$R_{eql}=\frac{\ln\frac{R_{2}}{R_{1}}}{2\pi \omega \varepsilon_{0}\varepsilon_{F}l_{l}}$$

(42) $$R_{eqr}=\frac{\ln\frac{R_{2}}{R_{1}}}{2\pi \omega \varepsilon_{0}\varepsilon_{F}l_{r}}$$

When the capacitance $C_{w}$ and the length of the seawater mixture are held constant, a quantitative investigation into the impact that various values of the dividing capacitance $C_{z}$ have on the measurement accuracy of capacitance $C_{w}$ is carried out. Since there is very little space between the cables that are located on the left and right sides of the U-shaped liquid level sensor, the length of the seawater mixture that is attached to the cables that are located on both sides is approximately the same, so $C_{eql}=C_{eqr}$ and $R_{eql}=R_{eqr}$ can be launched.

Take either value as the length of attached seawater mixture on the left and right sides, taking $l_{l}=l_{r}=10.0\;\mathrm{cm}$ as an example. $C_{w}=1.0\times 10^{-9}\;F$ is chosen as the theoretical value during simulation. Then, calculate the associated equivalent capacitance $C_{eql}=C_{eqr}=4.3943\times 10^{-11}\;F$ and equivalent resistance $R_{eql}=R_{eqr}=\begin{array}{c} 12072.7486 \end{array}\;\Omega$ using Formulas (39)–(42). Choose the value for regulating capacitance $C_{r}$ as $1.0\times 10^{-7}\;F$ in order to make the Formula $C_{w}+C_{r}\gg C_{eql}+C_{eqr}$ viable.

**Figure 7:**
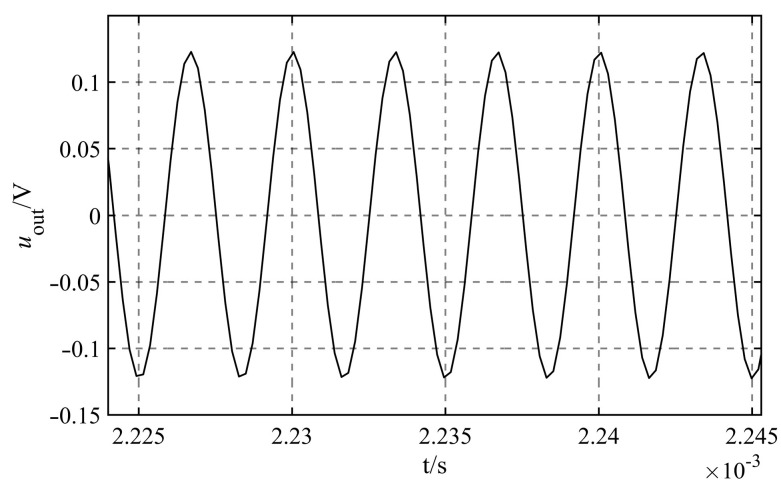
Output signals of $C_{z}=10^{-11}\;F$.

It is found that the capacitance less than $1.0\times 10^{-13}\;F$ or larger than $1.0\times 10^{-4}\;F$ is not common through the investigation of the capacitance circulating in the market. Considering the design of the signal processing circuit board for the liquid level sensor in the next step, in order to reduce the difficulty of maintenance and repair, it is recommended to choose common capacitor in the market for research. So the range of variation for the dividing capacitance $C_{z}$ is $1.0\times 10^{-13}\;F\leq C_{z}\leq 1.0\times 10^{-4}\;F$.Consider the values $C_{z}$ of the various orders of magnitude, as shown in Table 1, and simulate their effect on the measurement accuracy of capacitance $C_{w}$ under various scenarios. It can be found that the output signals under different circumstances are sinusoidal AC signals, taking the output signals of $C_{z}=1.0\times 10^{-11}\;F$ as an example, as shown in Figure 7.

The output signal $u_{\mathrm{out}}\left(t\right)$ that corresponds to the various dividing capacitance $C_{z}$ is fitted, and the fitted angular frequency is $1.885\times 10^{6}\;\mathrm{rad}/s$. As this value is consistent with the theoretical value of $\omega =2\pi f=1.885\times 10^{6}\;\mathrm{rad}/s$, it demonstrates that the angular frequency of the signal does not change at any point throughout the process of simulation. Table 1 displays the results of the fitting of the additional quantities.

The output signal’s function expression that corresponds to various dividing capacitance $C_{z}$ may be substituted into the calculation program of $C_{w}$. The output result and relative error are shown in Table 2. Based on the assumption that the length of the attached seawater mixture is constant, the capacitance $C_{w}=1.0\times 10^{-9}\;F$ and the regulating capacitance $C_{r}=1.0\times 10^{-7}\;F$, the inaccuracy in the simulated value of the capacitance $C_{w}$ in comparison to its theoretical value when the value of the dividing capacitance $C_{z}$ varies within the range of $10^{-14}-10^{-10}\;F$ and $10^{-8}-10^{-7}\;F$ is within ±1%. When the value of the dividing capacitance $C_{z}$ is $C_{z}=C_{w}=1.0\times 10^{-9}\;F$, the relative inaccuracy of the capacitance simulation value $C_{w}$ surpasses 8 percent. When the value of the dividing capacitance $C_{z}\geq 10^{-6}\;F$, the relative inaccuracy of the simulation value is greater than 3 percent, and the capacitance $C_{w}$ measurement accuracy is low.

After analyzing the value of the dividing capacitance $C_{z}$ is $C_{z}=C_{w}=1.0\times 10^{-9}\;F$, the reason why the relative inaccuracy of the simulated capacitance $C_{w}$ value is quite big is determined. According to Formulae (35)–(37), when the value of the dividing capacitance $C_{z}$ is $C_{z}=C_{w}$, the value of *X* is zero, while the value of *Y* is very tiny, and the order of magnitude $10^{-3}$ is typically. Hence, the output signal $u_{\mathrm{out}}\left(t\right)$ is now a faint signal with a very small amplitude. The acquisition of this weak signal is prone to produce a certain error. Thus, the relative inaccuracy of the capacitance $C_{w}$ computed by the algorithm is substantial. Consequently, $C_{z}=C_{w}$ should be avoided as much as feasible while selecting the dividing capacitance’s value.

The transformer bridge principle circuit has a detection capacitance goal range of 0−2000.0 pF, where the range of capacitance $C_{w}$ change is 0−2000.0 pF. Consequently, a value of the dividing capacitance $C_{z}$ that falls within the range of $0-2.0\times 10^{-9}\;F$ ought to be avoided at all costs. Furthermore, the relative inaccuracy of the capacitance values $C_{w}$ corresponding to the various $C_{z}$ values in Table 2 should be taken into account. The relative inaccuracy of the simulated value $C_{w}$ is quite substantial when the value of the dividing capacitance $C_{z}$ is within the range of $10^{-6}-10^{-4}\;F$, and the deviation value exceeds tens of picofarad. Because of this, it is important to avoid keeping the value of the dividing capacitance $C_{z}$ anywhere within the range of $10^{-6}-10^{-4}\;F$. The relative error corresponding to the dividing capacitance $C_{z}=1.0\times 10^{-7}\;F$ is smaller than the relative error corresponding to the dividing capacitance $C_{z}=1.0\times 10^{-8}\;F$, which means that the final value of the dividing capacitance is $C_{z}=1.0\times 10^{-7}\;F$.

### 5.2. The Influence of the Value of Regulating Capacitance $C_{r}$ on the Measurement Accuracy of Capacitance $C_{w}$

The approach of controlling a single variable is used in simulation studies to examine how the value of regulating capacitance $C_{r}$ affects the precision of capacitance measurements, and from there to establish the best possible value for regulating capacitance $C_{r}$.$C_{w}=1.0\times 10^{-9}\;F$ is chosen as the theoretical value during simulation. Additionally, Choose the dividing capacitance value as $C_{z}=1.0\times 10^{-7}\;F$. Take either value as the length of the attached seawater mixture on the left and right sides, taking $l_{l}=l_{r}=10.0\;\mathrm{cm}$ as an example. Then, calculate the associated equivalent capacitance $C_{eql}=C_{eqr}=4.3943\times 10^{-11}\;F$ and equivalent resistance $R_{eql}=R_{eqr}=\begin{array}{c} 12072.7486 \end{array}\Omega$ according to Equations (39)–(42).

**Figure 8:**
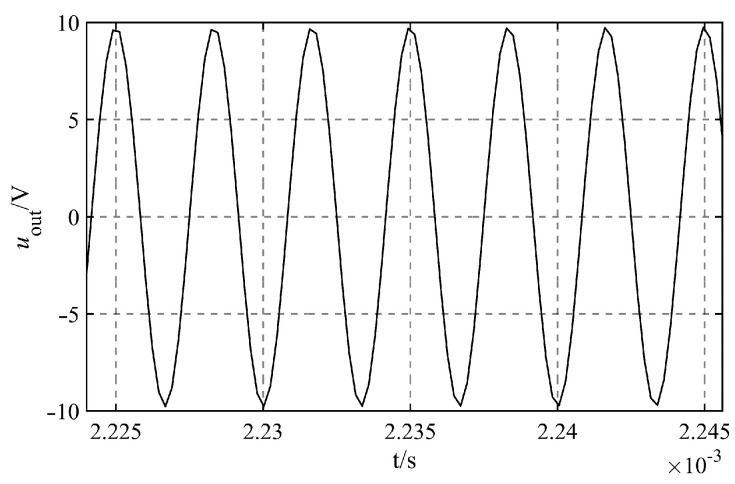
Output signals of $C_{r}=10^{-8}\;F$.

The value of the regulating capacitance $C_{r}$ must first satisfy the criteria of the formula $C_{w}+C_{r}\gg C_{eql}+C_{eqr}$, because the capacitance $C_{eql}+C_{eqr}=4.3943\times 10^{-11}\;F$, and capacitance $C_{w}$ is in the range of $0-2\times 10^{-9}\mathrm{pF}$, the value of $C_{r}$ cannot be less than $1.0\times 10^{-9}\;F$. It is found that the capacitance less than $1.0\times 10^{-13}\;F$ or larger than $1.0\times 10^{-4}\;F$ is not common through the investigation of the capacitance circulating in the market. Considering the design of the signal processing circuit board for the liquid level sensor in the next step, in order to reduce the difficulty of maintenance and repair, it is recommended to choose common capacitor in the market for research. So the range of variation for the regulating capacitance $C_{r}$ is $1.0\times 10^{-9}\;F\leq C_{r}\leq 1.0\times 10^{-4}\;F$. Under the premise that the guaranteed $C_{w}+C_{r}\gg C_{eql}+C_{eqr}$ is established, considering the values $C_{r}$ of the various orders of magnitude, as shown in Table 3. Additionally, simulate their effect on the measurement accuracy of capacitance $C_{w}$ under various scenarios. It can be found that the output signals under different circumstances are sinusoidal AC signals, taking the output signals of $C_{r}=1.0\times 10^{-8}\;F$ as an example, as shown in Figure 8.

.

The output signal $u_{\mathrm{out}}\left(t\right)$ that corresponds to the various regulating capacitances $C_{r}$ is fitted, and the fitted angular frequency is $1.885\times 10^{6}\;\mathrm{rad}/s$. Since this value is consistent with the theoretical value of $\omega =2\pi f=1.885\times 10^{6}\;\mathrm{rad}/s$, it demonstrates that the angular frequency of the signal remains unchanging throughout the process of simulation. Table 3 displays the fitting outcomes for all other variables.

Table 4 demonstrates the output result and relative error achieved by substituting the function expression of the output signal corresponding to the various regulating capacitances $C_{r}$ into the $C_{w}$ calculation program. Under the assumption that the length of the attached seawater mixture is fixed, the capacitance is $C_{w}=1.0\times 10^{-9}\;F$, and the dividing capacitance is $C_{z}=1.0\times 10^{-7}\;F$, it is demonstrates that when the value of regulating capacitance $C_{r}$ changes within the range of $10^{-9}-10^{-7}\;F$, the error of $C_{w}$ the simulated capacitance value relative to the theoretical value is within ±1%. Nevertheless, when the value of the regulating capacitance varies within the range of $10^{-6}-10^{-5}\;F$, the relative inaccuracy of the simulation value is relatively larger. When the value of regulating capacitance $C_{r}$ is $10^{-4}\;F$, there is a significant deviation of the simulation value $C_{w}$.

When the value of the regulating capacitance $C_{r}$ is $10^{-4}\;F$, analyze the cause of the simulation value’s error $C_{w}$. Since the output capacitor $C_{w}$ and regulating capacitance $C_{r}$ share the phase voltage $\overset{\cdot }{E_{1}}$, if the value of the regulating capacitance $C_{r}$ is too high, the regulating capacitance will nearly completely share the phase voltage. At this point, the output signal $u_{\mathrm{out}}\left(t\right)$ will scarcely be affected by a change in capacitance $C_{w}$. In accordance with Formulas (35)–(37), the output signal $u_{\mathrm{out}}\left(t\right)$ at this time is a weak signal with a very small amplitude, and the error of the weak signal in fitting is large. This directly results in a serious deviation of the calculation results of capacitance $C_{w}$ through the following program. So there is an upper limit to the value of regulating capacitance $C_{r}$.The value of the regulating capacitance $C_{r}$ should not be larger than $1.0\times 10^{-4}\;F$, however, since from the standpoint of practical application, if it is, the weak signal $u_{\mathrm{out}}\left(t\right)$ supplied by the circuit is particularly sensitive to outside interference and immersed in noise in practical use.

According to the simulation results in Table 4, when the capacitance $C_{w}=1.0\times 10^{-9}\;F$ and $C_{eql}+C_{eqr}=4.3943\times 10^{-11}\;F$, and the value of the regulating capacitance $C_{r}$ is within the range of $10^{-9}-10^{-7}\;F$, the accuracy of the simulated value of the capacitance $C_{w}$ is high. It shows that when the value of $C_{w}+C_{r}$ is 2 to 5 orders of magnitude larger than the value of $C_{eql}+C_{eqr}$, the formula $C_{w}+C_{r}\gg C_{eql}+C_{eqr}$ is established, and the precision of the simulation value of capacitance $C_{w}$ is higher. When $C_{r}=1.0\times 10^{-8}\;F$, $C_{w}+C_{r}=1.1\times 10^{-8}\;F$, $C_{w}$ have the highest accuracy of simulation value, which is only 0.01%. It is indicated that when $C_{w}+C_{r}$ is 3 orders of magnitude larger than $C_{eql}+C_{eqr}$, $C_{w}$ has the highest accuracy of simulation value.

The spectrum of capacitance $C_{w}$ is $0-2\times 10^{-9}\;\mathrm{pF}$. Furthermore, according to Formulas (39) and (40), the equivalent capacitance $C_{eql}$ and $C_{eqr}$ rise as the length of the attached seawater mixture grow. When the length of the attached seawater mixture is 0.005 m, the equivalent capacitance $C_{eql}$ and $C_{eqr}$ has an order of magnitude of −12. When the length of the attached seawater mixture is 0.03 m, the equivalent capacitance $C_{eql}$ and $C_{eqr}$ has an order of magnitude of −11. When the length of the attached seawater mixture is 0.23 m, the equivalent capacitance $C_{eql}$ and $C_{eqr}$ has an order of magnitude of −10. Applying the concept of limits, when the length of the attached seawater mixture is larger than 0.23 m and less than 2.020 m, the order of magnitude of the equivalent capacitance $C_{eql}$ and $C_{eqr}$ remains at −10, but it should be apparent that the real length of the attached seawater mixture will not exceed 2.020 m.

To establish the formula $C_{w}+C_{r}\gg C_{eql}+C_{eqr}$ and ensure greater measurement accuracy of capacitance $C_{w}$, it is necessary to make the value $C_{w}+C_{r}$ 2 to 5 orders of magnitude greater than the value of $C_{eql}+C_{eqr}$. As the equivalent capacitance of different lengths of attached seawater mixture is predominantly between −11 and −10, and to determine the value of the regulating capacitance as $C_{r}=1.0\times 10^{-7}\;F$.

### 5.3. Circuit Parameters

The simulation and analysis of the output signal $u_{\mathrm{out}}\left(t\right)$ for varying values of dividing capacitance $C_{z}$ and regulating capacitance $C_{r}$ are performed using the single variable research method. The fitting results of the output signal $u_{\mathrm{out}}\left(t\right)$ under varying conditions and the relative error of capacitance $C_{w}$ are examined. Finally, $C_{z}=1.0\times 10^{-7}\;F$ and $C_{r}=1.0\times 10^{-7}\;F$ are determined. The important characteristics of the circuit based on the transformer bridge concept are listed in Table 5.

## 6. Simulation and Verification of Transformer Bridge Principle Circuit

### 6.1. Simulation Results and Analysis When the Capacitance $C_{w}$ Changes

In order to validate the efficiency of the transformer principle bridge circuit in reducing the lag stability of the capacitance value of the U-shaped liquid level sensor, the transformer principle bridge circuit’s characteristics are primarily evaluated from two perspectives. On the one hand, calculating the accuracy of the capacitance $C_{w}$ simulation value obtained through transformer principle bridge circuit and corresponding program when the capacitance $C_{w}$ changes. On the other hand, when the length of the attached seawater mixture varies, measuring the accuracy of $C_{w}$ simulation value. If the simulated value of capacitance $C_{w}$ obtained from measurement has high accuracy, it will verify that the transformer principle bridge circuit has the capability to effectively reduce the lag stability of the U-shaped liquid level sensor’s capacitance value. Instead, if the measurement accuracy is low, it will indicate that the principle circuit is not suitable for the study of reducing the stability impact of output capacitance hysteresis.

For simulation verification, the method of controlling a single variable is utilized. The length of the attached seawater mixture is maintain a certain value, the value of capacitance $C_{eql}$, capacitance $C_{eqr}$, resistance $R_{eql}$ and resistance $R_{eqr}$ maintain a fixed value. Calculating the measurement precision when the capacitance $C_{w}$ varies. The length of the seawater mixture attached to the cables on the left and right sides should be taken as one value. As an example $l_{l}=l_{r}=10.0\;\mathrm{cm}$, the relevant equivalent capacitance $C_{eql}=C_{eqr}=4.3943\times 10^{-11}\;F$ and equivalent resistance $R_{eql}=R_{eqr}=\begin{array}{c} 12072.7486\; \end{array}\Omega$ are computed using Formulas (39)–(42).

**Figure 9:**
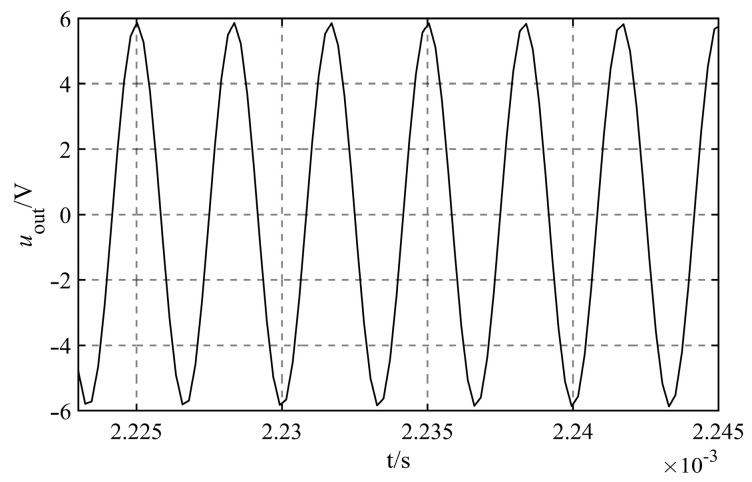
Output signal of $C_{w}=1.0\times 10^{-9}\;F$.

Specifically, the regulating capacitance is $C_{r}=1.0\times 10^{-7}\;F$, and the dividing capacitance is $C_{z}=1.0\times 10^{-7}\;F$. The transformer bridge principle circuit has a capacitance detection target range of 0−2000.0 pF. Table 6 demonstrates that the changing gradient of capacitance $C_{w}$ should be set at 200 pF. Simulate the measurement accuracy as a function of the capacitance $C_{w}$ change. It can be found that the output signals under different circumstances are sinusoidal AC signals, taking the output signals of $C_{w}=1.0\times 10^{-9}\;F$ as an example, as shown in Figure 9.

The output signal $u_{\mathrm{out}}\left(t\right)$ that corresponds to the various capacitances $C_{w}$ is fitted, and the fitted angular frequency is $1.885\times 10^{6}\;\mathrm{rad}/s$. Since this value is consistent with the theoretical value of $\omega =2\pi f=1.885\times 10^{6}\;\mathrm{rad}/s$, it demonstrates that the angular frequency of the signal remains unchanging throughout the process of simulation. The results of fitting other quantities are shown in Table 6.

After the fitting process, the equations for the function of the output signal that corresponds to the various capacitances $C_{w}$ are derived and then replaced into the calculation program. The simulated value of capacitance $C_{w}$, as well as its relative error, may be apparent in Table 7. When the length of the attached seawater mixture is kept fixed, the relative error of the simulated capacitance $C_{w}$ is contained within ±1%, and the precision of the measurement is good. It demonstrates that the transformer principle bridge circuit can effectively reduce the lag stability of the capacitance value of the U-shaped liquid level sensor when the length of the attached seawater mixture is fixed.

### 6.2. Simulation Results and Analysis When the Length of Attached Seawater Mixture Changes

**Figure 10:**
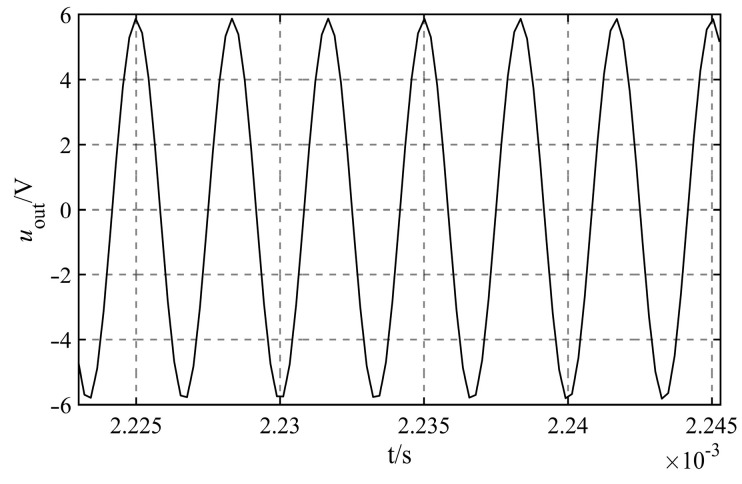
Output signal of $l_{l}=l_{r}=8\;\mathrm{cm}$.

For simulation verification, the approach of manipulating a single variable is utilized. The detecting capacitance goal range of the transformer bridge principle circuit is 0−2000.0 pF, and the target range’s intermediate value of 1000.0 pF is selected as the capacitance value $C_{w}$ during simulation. The dividing capacitance is $C_{z}=1.0\times 10^{-7}\;F$ and the regulating capacitance is $C_{r}=1.0\times 10^{-7}\;F$. Simulate the measurement accuracy of the capacitance $C_{w}$ when the length $l_{l}$ and $l_{r}$ of the seawater mixture attached to the cables on the left and right sides of the U-shaped liquid level sensor have different values. Using $l_{l}$ and $l_{r}$ as distinct values, compute the associated equivalent capacitance $C_{eql}=C_{eqr}$ and equivalent resistance $R_{eql}=R_{eqr}$ using Formulae (39)–(42), as given in Table 8. $l_{l}=l_{r}=0.5\;\mathrm{cm}$ represents the scenario in which a very short length of the attached seawater mixture is present. As shown in Figure 10, the relevant output signal $u_{\mathrm{out}}\left(t\right)$ under various situations is generated via simulation calculation. It can be seen that the output signal is a sinusoidal AC signal, taking the output signals of $l_{l}=l_{r}=8\;\mathrm{cm}$ as an example, as shown in Figure 10.

The output signal $u_{\mathrm{out}}\left(t\right)$ corresponding to the length of different attached seawater mixtures is fitted, and the fitted angular frequency is $1.885\times 10^{6}\;\mathrm{rad}/s$, which is consistent with the theoretical value of angular frequency $\omega =2\pi f=1.885\times 10^{6}\;\mathrm{rad}/s$, demonstrating that the signal’s angular frequency is always steady throughout the simulation process. Table 9 displays the fitting results of various quantities.

The output signal $u_{\mathrm{out}}\left(t\right)$ function obtained by fitting is substituted into the calculation program of $C_{w}$. Table 10 displays the output result and relative error. As shown in Table 10, the relative error of capacitance $C_{w}$ under various situations is within ±1%, and the precision of the measurement is excellent. It demonstrates that the transformer principle bridge circuit can effectively reduce the lag stability of the capacitance value of the U-shaped liquid level sensor induced by the attached seawater mixture when the length of the attached seawater mixture varies.

## 7. Conclusions

To reduce impact of the lag stability of the capacitance value during the level drop, a signal processing method that uses the phase difference was proposed on the basis of analyzing the equivalent circuit of the dirty U-shaped liquid level sensor, and a signal processing principle verification circuit was designed. Additionally, the signal processing circuit was then simulated by MATLAB Simulink software, the values of dividing capacitance and regulating capacitance were determined, and the effectiveness of the signal processing circuit in reducing the influence of output capacitance hysteresis stability was verified. The following is an outline of the main contents.

(1) When the length of the attached seawater mixture is long enough, it can be concluded mathematically that the impedance modulus of the equivalent resistance and the equivalent capacitance are identical to one another. The circuit model of a U-shaped liquid level sensor with dirt is constructed on the basis of this information.

(2) A design was developed for the fundamental circuit of a transformer bridge, which can get rid of the sensor’s lag stability. It is inferred that the output signal $u_{\mathrm{out}}\left(t\right)$ of the circuit is integrated in the phase range π/4−5π/4, which may remove the impact of the attached seawater mixture, and the capacitance $C_{w}$ can be computed based on the definitive integral result of the output signal $u_{\mathrm{out}}\left(t\right)$.

(3) The fundamental circuit of the transformer bridge is simulated using the MATLAB Simulink software, and the values of the dividing capacitance $C_{z}$ and the regulating capacitance $C_{r}$ are controlled by changing a single variable. On the basis of this, individually regulate the change in capacitance $C_{w}$ and the change in the length of the attached seawater mixture $l_{l}$ and $l_{r}$, and then simulate and compute the simulated value of the capacitance $C_{w}$ under various situations. As the relative error ±1% is within and the accuracy is excellent, this demonstrates that the transformer principle bridge circuit is successful in getting rid of the lag stability of the capacitance value of the U-shaped liquid level sensor.

## Figures and Tables

**Table 1:** Fitting results of output signals corresponding to different dividing capacitance.

$C_{z}$ **(F)**	***a*** **_0_**	***a*** **_1_**	***b*** **_1_**	**SSE**
$1.0\times 10^{-13}$	$2.785\times 10^{-4}$	0.124	0.005163	$1.181\times 10^{-6}$
$1.0\times 10^{-12}$	$-1.327\times 10^{-4}$	0.1239	0.005159	$1.496\times 10^{-14}$
$1.0\times 10^{-11}$	$-1.292\times 10^{-6}$	0.1228	0.005159	$9.279\times 10^{-9}$
$1.0\times 10^{-10}$	$-1.671\times 10^{-4}$	0.112	0.005159	$1.656\times 10^{-14}$
$1.0\times 10^{-9}$	$-2.45\times 10^{-5}$	−0.006591	0.00311	$3.012\times 10^{-4}$
$1.0\times 10^{-8}$	$-4.719\times 10^{-4}$	−0.967	0.005255	$1.759\times 10^{-4}$
$1.0\times 10^{-7}$	$-4.225\times 10^{-5}$	−5.877	0.004717	$9.566\times 10^{-14}$
$1.0\times 10^{-6}$	$2.335\times 10^{-5}$	−10.78	0.005071	$7.323\times 10^{-14}$
$1.0\times 10^{-5}$	$8.722\times 10^{-4}$	−11.76	0.005361	$9.464\times 10^{-14}$
$1.0\times 10^{-4}$	$-2.504\times 10^{-5}$	−11.86	0.005232	$1.601\times 10^{-13}$

**Table 2:** Output results and relative errors corresponding to different dividing capacitance.

**Theoretical Value of** $\;C_{w}$ **(F)**	$C_{z}$ **(F)**	**Simulation Value of** $\;C_{w}$ **(F)**	**Relative Error**
$1.0\times 10^{-9}$	$1.0\times 10^{-13}$	$9.9506\times 10^{-10}$	−0.49%
	$1.0\times 10^{-12}$	$1.0029\times 10^{-9}$	0.29%
	$1.0\times 10^{-11}$	$1.0003\times 10^{-9}$	0.03%
	$1.0\times 10^{-10}$	$1.0033\times 10^{-9}$	0.33%
	$1.0\times 10^{-9}$	$9.1806\times 10^{-10}$	−8.19%
	$1.0\times 10^{-8}$	$1.0076\times 10^{-9}$	0.76%
	$1.0\times 10^{-7}$	$9.9630\times 10^{-10}$	−0.37%
	$1.0\times 10^{-6}$	$1.0439\times 10^{-9}$	4.39%
	$1.0\times 10^{-5}$	$9.5817\times 10^{-10}$	−4.18%
	$1.0\times 10^{-4}$	$1.0342\times 10^{-9}$	3.42%

**Table 3:** Fitting results of output signals corresponding to different regulating capacitance.

$C_{r}$ **(F)**	***a*** **_0_**	***a*** **_1_**	***b*** **_1_**
$1.0\times 10^{-9}$	$4.034\times 10^{-3}$	−5.749	0.126
$1.0\times 10^{-8}$	$4.899\times 10^{-4}$	−9.774	0.04315
$1.0\times 10^{-7}$	$-4.225\times 10^{-5}$	−5.877	0.004717
$1.0\times 10^{-6}$	$8.329\times 10^{-9}$	−1.078	$5.24\times 10^{-4}$
$1.0\times 10^{-5}$	$-5.803\times 10^{-7}$	−0.1176	$5.269\times 10^{-5}$
$1.0\times 10^{-4}$	$-1.068\times 10^{-7}$	−0.01186	$-2.031\times 10^{-4}$

**Table 4:** Output results and relative errors corresponding to different regulating capacitance.

**Theoretical Value of** $\;C_{w}$ **(F)**	$C_{r}$ **(F)**	**Simulation Value of** $\;C_{w}$ **(F)**	**Relative Error**
$1.0\times 10^{-9}$	$1.0\times 10^{-9}$	$1.0018\times 10^{-9}$	0.18%
	$1.0\times 10^{-8}$	$9.9994\times 10^{-10}$	−0.01%
	$1.0\times 10^{-7}$	$9.9630\times 10^{-10}$	−0.37%
	$1.0\times 10^{-6}$	$1.0332\times 10^{-9}$	3.32%
	$1.0\times 10^{-5}$	$9.6716\times 10^{-10}$	−3.28%
	$1.0\times 10^{-4}$	$2.7613\times 10^{-9}$	176.13%

**Table 5:** Parameters of the simulated circuit.

Parameter	Value	Unit
$E_{0}$	24	V
f	300,000	Hz
$E_{1}$	12	V
$C_{z}$	$1.0\times 10^{-7}$	F
$C_{r}$	$1.0\times 10^{-7}$	F

**Table 6:** Fitting results of output signals corresponding to different capacitances $C_{w}$.

$C_{w}$ **(F)**	***a*** **_0_**	***a*** **_1_**	***b*** **_1_**
0	$3.76\times 10^{-11}$	−5.995	0.005256
$2.0\times 10^{-10}$	$-8.941\times 10^{-5}$	−5.971	0.005382
$4.0\times 10^{-10}$	$-1.987\times 10^{-4}$	−5.947	0.00535
$6.0\times 10^{-10}$	$1.385\times 10^{-4}$	−5.923	0.005131
$8.0\times 10^{-10}$	$3.969\times 10^{-4}$	−5.899	0.005026
$1.0\times 10^{-9}$	$-4.225\times 10^{-5}$	−5.877	0.004717
$1.2\times 10^{-9}$	$-1.631\times 10^{-4}$	−5.853	0.005155
$1.4\times 10^{-9}$	$-5.913\times 10^{-4}$	−5.83	0.005216
$1.6\times 10^{-9}$	$4.451\times 10^{-5}$	−5.806	0.005028
$1.8\times 10^{-9}$	$8.47\times 10^{-5}$	−5.782	0.004865
$2.0\times 10^{-9}$	$3.712\times 10^{-4}$	−5.759	0.004943

**Table 7:** Output results and relative errors corresponding to different output capacitances.

$C_{z}$ **(F)**	$C_{r}$ **(F)**	**Theoretical Value of ** $C_{w}$ **(F)**	**Simulation Value of ** $C_{w}$ **(F)**	**Relative Error**
$1.0\times 10^{-7}$	$1.0\times 10^{-7}$	0	−2.1333	--
		$2.0\times 10^{-10}$	$1.9987\times 10^{-10}$	−0.07%
		$4.0\times 10^{-10}$	$4.0237\times 10^{-10}$	0.59%
		$6.0\times 10^{-10}$	$5.9992\times 10^{-10}$	−0.01%
		$8.0\times 10^{-10}$	$7.9877\times 10^{-10}$	0.15%
		$1.0\times 10^{-9}$	$9.9630\times 10^{-10}$	−0.37%
		$1.2\times 10^{-9}$	$1.1993\times 10^{-9}$	−0.06%
		$1.4\times 10^{-9}$	$1.4036\times 10^{-9}$	0.26%
		$1.6\times 10^{-9}$	$1.5991\times 10^{-9}$	−0.06%
		$1.8\times 10^{-9}$	$1.8066\times 10^{-9}$	0.37%
		$2.0\times 10^{-9}$	$1.9995\times 10^{-9}$	−0.03%

**Table 8:** Equivalent capacitance and equivalent resistance corresponding to different lengths.

*l_l_ = l_r_* (cm)	$C_{eql}=C_{eqr}$(F)	$R_{eql}=R_{eqr}$(Ω)
0.5	$2.1972\times 10^{-12}$	241,454.9725
4.0	$1.7577\times 10^{-11}$	30,181.8716
8.0	$3.5155\times 10^{-11}$	15,090.9358
12	$5.2732\times 10^{-11}$	10,060.6239
16	$7.0309\times 10^{-11}$	7,544,679
20	$8.7887\times 10^{-11}$	6036.3743
24	$1.0546\times 10^{-10}$	5030.3119
28	$1.2304\times 10^{-10}$	4311.6959
32	$1.4062\times 10^{-10}$	3772.7339
36	$1.5820\times 10^{-10}$	3353.5413

**Table 9:** Fitting results of output signals corresponding to different lengths of attached seawater mixture.

$l_{l}=l_{r}$ **(cm)**	***a*** **_0_**	***a*** **_1_**	***b*** **_1_**
0.5	$-2.228\times 10^{-4}$	−5.881	$3.103\times 10^{-4}$
4.0	$1.136\times 10^{-4}$	−5.879	$1.996\times 10^{-3}$
8.0	$2.384\times 10^{-5}$	−5.877	$4.166\times 10^{-3}$
12	$3.493\times 10^{-5}$	−5.875	$6.208\times 10^{-3}$
16	$1.422\times 10^{-4}$	−5.872	$8.637\times 10^{-3}$
20	$1.236\times 10^{-4}$	−5.87	$1.069\times 10^{-2}$
24	$1.635\times 10^{-4}$	−5.869	$1.228\times 10^{-2}$
28	$1.554\times 10^{-4}$	−5.866	$1.479\times 10^{-2}$
32	$4.135\times 10^{-4}$	−5.864	$1.637\times 10^{-2}$
36	$-1.303\times 10^{-4}$	−5.863	$1.852\times 10^{-2}$

**Table 10:** Output results and relative errors corresponding to different lengths of attached seawater mixture.

$l_{l}=l_{r}(\mathrm{cm})$	$C_{w}$ **(F)**	**Simulation Value of** $\;C_{w}$ **(F)**	**Relative Error**
0.5	$1.0\times 10^{-9}$	$1.0032\times 10^{-9}$	0.32%
4.0		$9.9949\times 10^{-10}$	−0.05%
8.0		$9.9974\times 10^{-10}$	−0.03%
12		$9.9917\times 10^{-10}$	−0.08%
16		$1.0020\times 10^{-9}$	0.20%
20		$1.0019\times 10^{-9}$	0.19%
24		$9.9613\times 10^{-10}$	−0.39%
28		$1.0004\times 10^{-9}$	0.04%
32		$9.9915\times 10^{-10}$	−0.09%
36		$9.9964\times 10^{-10}$	−0.04%

## Data Availability

Not applicable.
